# Robust Control Strategy of Gradient Magnetic Drive for Microrobots Based on Extended State Observer

**DOI:** 10.34133/2022/9835014

**Published:** 2022-10-21

**Authors:** Jiawei Lu, Yueyue Liu, Wentao Huang, Kaitao Bi, Yixin Zhu, Qigao Fan

**Affiliations:** College of Internet of Things Engineering, Jiangnan University, Wuxi 214000, China

## Abstract

Microrobots have great application potential in the biomedical field, to realize the precision and efficiency of microrobots in vivo is research focus in this field. Microrobots are accompanied by various disturbances in complex environment. These disturbances will affect the motion control of microrobots, resulting in the inability of the micromanipulation tasks to be completed effectively. To this end, a robust motion control method is proposed for precise path tracking of microrobots in this paper. The extended state observer (ESO) is used to estimate the total disturbances and uncertainties of the system. A path tracking controller is designed by combining sliding mode control (SMC) and disturbances compensation, which is used to eliminate the total disturbances of the system and realize the fast and accurate path tracking of microrobots. Finally, the path tracking experiments are implemented in the gradient magnetic field drive system. The experimental results show that the mean absolute error of the path tracking for microrobots in a simulated vascular structure is less than 14 *μ*m, and the root mean square error is less than 17 *μ*m by using the robust control method proposed in this paper. Compared with the traditional PID control method, it can better suppress external disturbances and uncertainties of the system and improve the path tracking accuracy of microrobots effectively. It shows stronger anti-interference ability and robustness.

## 1. Introduction

Microrobots have become the micromanipulation objects suitable for biomedicine due to their unique properties that can be remotely manipulated in recent years. Because of their small size, microrobots can operate in vivo at the micro-nanoscale [1]. There are many driving methods for microrobots. It is worth noting that the magnetic field-based driving methods [2–4] have great advantages in operating microrobots in vivo, mainly because the magnetic fields generated by the energized coils have a strong permeability in the biological tissue. Moreover, most living organisms are not sensitive to magnetic fields, that is, the magnetic fields are nondestructive to living organisms [5]. At present, magnetic microrobots are widely used in biomedicine, such as targeted drug delivery, cell micromanipulation, and vascular dredging [6–9].

The magnetic fields driving methods of microrobots are generally divided into rotating magnetic field, oscillating magnetic field, and gradient magnetic field [10–13]. The rotating magnetic field and oscillating magnetic field driving methods usually require the microrobots to be designed into a specific structure to generate the driving force. However, it will complicate the structural design of the microrobots and reduce the control accuracy [14]. The driving method based on gradient magnetic field can directly provide magnetic force to the microrobots and make it move [15]. This driving method has no special requirements for the shape of magnetic microrobots. Gradient magnetic field can be generated by permanent magnets, Maxwell coils, and electromagnetic coils [16, 17]. Permanent magnets can perform control tasks by adjusting the end effectors of the manipulator attached to it [18]. This method is more complicated and requires a large movable space. Maxwell coils can generate constant gradient magnetic field, but the gradient is usually small and cannot drive microrobots in complex environment or in vivo effectively. The electromagnetic coils are composed of a soft magnetic core and coils. The addition of the soft magnetic core can enhance the magnetic field and generate a stronger magnetic field gradient, making it possible for the microrobots to be driven in a living environment. In this paper, we will research the motion control of microrobots based on the gradient-enhanced electromagnetic drive system.

Several papers have reported the use of gradient magnetic field for motion control of microrobots in complex environment. An enhanced electromagnetic operating system with a large working space was developed, which realized the trajectory tracking control experiments of magnetic particles in two-dimensional and three-dimensional space by using proportional integral derivative (PID) controller [19]. A model-based nonlinear controller was designed, it can realize real-time visual tracking and position control of microbeads in a three-dimensional workspace [20]. A controller based on the feedback of Optical Coherence Tomography (OCT) was developed and realized the automatic navigation experiments of the microrobot in mice on a gradient magnetic field driven platform [21]. In other magnetic field driving fields of microrobot motion control [22], a control structure composed of feedforward controller and feedback fuzzy logic controller was designed, which can automatically adjust the pattern deformation, orientation, and position of the magnetic microrobot swarm. An adaptive control method of magnetically levitated microrobot based on augmented error was proposed [23]. Simulation and experiment show that the adaptive control algorithm can improve the system performance. In [24], a motion control method for intravascular navigation of microrobot based on model predictive controller was designed. Simulation results show the effectiveness of the proposed algorithm. It is worth mentioning that these control methods were designed under ideal conditions, the uncertainties of system parameters and external environmental disturbances have not been fully considered and dealt with effectively, which will have an impact on the motion control of microrobot, so that a satisfactory tracking effect cannot be achieved. Therefore, it has become an urgent need to design a robust control method to achieve precise control of microrobots in complex environment.

It is reported that the problem of disturbances and uncertainties of system parameters in complex environment can be solved by the observers. Observers can effectively estimate the total disturbances of the system, and the total disturbances can be compensated by designing the controller to improve the motion control accuracy of microrobots. This kind of observers mainly include sliding mode observer [25], high gain observer [26], and extended state observer [27, 28]. A navigation control method of microrobot based on adaptive backstepping law and high gain observer was designed in [29]. The results show that the average tracking error of the microrobot with a diameter of 500 *μ*m is about 100 *μ*m. Simulations and experiment illustrate the robustness to both noise measurement and some uncertain physiological parameters. For the servo motor system, a high-order sliding mode observer was used to estimate the unknown state of the transformed system, and the adaptive controller was used to realize the tracking control. When tracking the sinusoidal curve with a period of 2 s, the integral absolute error of the proposed control method is 11.78 degrees, which is reduced by more than 50% compared with the PID control [25]. In [26], an output feedback control method based on a high gain observer was proposed to realize the trajectory tracking control of ROVs with four degrees of freedom when the system had unmodeled equipment dynamics, parameter changes, measurement errors, noise, and environmental interference. The experimental results show that it can successfully track the plane path in the horizontal plane with a mean error of 37.6 cm by using the proposed control method, achieving a satisfactory tracking performance. In [27], using ESO to estimate uncertainties from internal parametric models, unknown fluid dynamics, and external disturbances due to wind, waves, and ocean currents, the results confirmed the effectiveness of the proposed ESO in state recovery and uncertainties estimation for unmanned surface vehicles. A disturbance rejection control strategy based on ESO was proposed [28]. In the hover experiment of the interference with wind speed of 6 m/s, compared without ESO, the quadrotor vehicle achieve about 57% reduction on the root mean square error of trajectory tracking error and achieve about 59% and 22% reductions on the root mean square error of velocity and attitude tracking error, respectively. Meanwhile, the circular trajectory tracking experiments can prove that the control strategy can successfully achieve high-precision trajectory tracking of the quadrotor vehicle with disturbances caused by the wind gust and ground effect.

Aiming at the disturbances and system uncertainties of microrobots in complex environment, a robust control method based on ESO is proposed in this paper. The uncertainties of system parameters and external environmental disturbances are regarded as the total disturbances, which can be obtained by the ESO. Then, a path tracking controller is designed by combining sliding mode control and disturbances compensation to eliminate the total disturbances of the system and ensure the robustness of the tracking. Finally, the path tracking control experiments of microrobot is carried out in a simulated vascular structure. The results show that the control method proposed in this paper can improve path tracking accuracy and solve the disturbances problem of the microrobot in a complex environment.

This paper is organized as follows: Section 2 presents the magnetic and dynamic models of microrobot. Designing ESO and the path tracking control of microrobot is illustrated in Section 3. Experimental investigation of the above-mentioned method is presented in Section 4. Finally, a conclusion is given in Section 5.

## 2. Dynamic Modeling of Microrobot

### 2.1. Magnetic Model

The magnetic microrobot is placed in gradient magnetic field will generate a magnetic force *F*_mag_. The magnetic force of the microrobot in a magnetic field with magnetic flux density *B* can be expressed as follows [30]:

(1) $$\begin{array}{c} {\text{F}}_{\text{mag}}=\text{TV}\left(\text{M}\times \nabla \right)\text{B}, \end{array}$$

where *T* is the ratio of the volume of magnetic material to the volume of the microrobot, *V* is the volume of the microrobot, and *M* is the magnetization of the microrobot.

According to Equation (1), the key to obtain the magnetic force of microrobot is to calculate the magnetic flux density *B*. The magnetic field generated by the electromagnetic coils in the workspace is calculated below. The magnetic flux density *B* generated by the electromagnetic coils system is determined by the current *I*. In the research process, the current of the electromagnetic coils has an approximately linear relationship with the magnetic flux density and gradient; thus, the magnetic flux density *B* and the magnetic field gradient ∇*B* generated by a single electromagnetic coil can be expressed as

(2) $$\begin{array}{c} \left\{\begin{array}{c} B=B^{\prime }I,\\ \nabla B=\nabla B^{\prime }I, \end{array}\right. \end{array}$$

where ∇*B*′ is the unit magnetic field gradient generated by the energized coils and *B*′ is the unit magnetic flux density generated by the energized coils. *B*′ can be approximately calculated by the point dipole model [1]

(3) $$\begin{array}{c} B^{\prime }\left(\Gamma ,P\right)=\frac{\mu_{0}}{4\pi {\left|P\right|}^{3}}\left(\frac{3\left(\Gamma \cdot P\right)P}{{\left|P\right|}^{2}}-\Gamma \right), \end{array}$$

where $\mu_{0}=4\pi \times 10^{-7}Tm/A$ is the permeability of free space, *P* = [*P*_*x*_, *P*_*y*_]^*T*^ is the position of the microrobot in the workspace, and Γ represents a point dipole.

Consequently, the magnetic force *F*_mag_ generated by the magnetic microrobot placed in the gradient magnetic field has a certain functional relationship with the current *I* of electromagnetic coils and the position *P* of the microrobot.

### 2.2. Dynamic Modeling of Microrobot

The motion of microrobot in liquid environment will be affected by the resistance of solution. The spherical magnetic microrobot was used in the research process, and the moving medium solution of the microrobot has low Reynolds number. According to [31], the viscous resistance of the microrobot is

(4) $$\begin{array}{c} F_{\text{drag}}=6\pi \eta Rv, \end{array}$$

where *R* is the radius of the spherical magnetic microrobot, *η* represents the dynamic viscosity of the fluid, and *v* represents the moving velocity of the microrobot.

Microrobot will be affected by magnetic force, viscous resistance, gravity, buoyancy, friction, and other forces in the low Reynolds number liquid environment. For the microrobot moving in two-dimensional space, its gravity is equal to the sum of buoyancy and platform support force. Therefore, the dynamic equation of the microrobot is written as follows:

(5) $$\begin{array}{c} m\ddot{P}=F_{\text{mag}}-F_{\text{drag}}+\Delta . \end{array}$$

where *m* is the mass of the magnetic microrobot. In actual operation, the system is vulnerable to other external disturbances, including magnetic field noise, external environment vibration, friction, other unknown forces, and the uncertainties of the system model. These disturbances will affect the motion control of the magnetic microrobot and reduce the control accuracy. Therefore, adding the above disturbances to Δ, which can be handled centrally by designing controller.

## 3. Motion Control

Microrobot will be subject to various disturbances in complex environment, which will affect the motion control accuracy of microrobot. To deal with the disturbances in the path tracking control process of microrobot, two aspects should be considered:

1. The real-time position of the microrobot can be obtained by camera, but the total disturbances cannot be measured directly, so ESO needs to be designed to observe it
2. A controller combining sliding mode control and disturbances compensation is designed to eliminate the total disturbances and suppress the tracking errors to ensure the robustness of path tracking

### 3.1. Path Tracking

**Figure 1:**
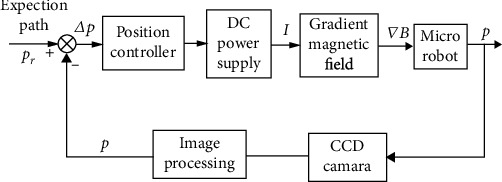
Microrobot path tracking control.

The path tracking control block diagram of the microrobot is shown in Figure 1:

The path tracking process of the microrobot can be briefly summarized as follows: firstly, a desired path is designed; then, the actual position of the microrobot is obtained through the CCD camera. The position deviation of the microrobot is processed by the position controller, which can control the current passed through the electromagnetic drive system to control the motion until the microrobot reaches the desired position.

**Figure 2:**
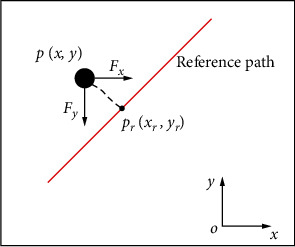
Path tracking analysis.

Considering that the electromagnetic drive system has a two-dimensional workspace, and the system is composed of two pairs of orthogonal electromagnetic coils with the same parameters. Thus, the motion control of the microrobot in the two-dimensional workspace can be simply divided into two directions of independent control. The position error of the microrobot can be decomposed into the error in *x* and *y* directions, as shown in Figure 2. The position error in the *x*-axis is handled by the electromagnetic coils in the *x*-axis, and the microrobot is driven to the desired position *x*_*r*_ by the magnetic force in the *x*-direction. Similarly, the position error in the *y*-axis is handled by the electromagnetic coils in the *y*-axis, and the microrobot is driven to the desired position *y*_*r*_ by the magnetic force in the *y*-direction. Finally, the path tracking of microrobot is realized by the interaction of electromagnetic coils in two directions.

### 3.2. Design of ESO

In the process of path tracking control, the position of the microrobot is expressed by *p*, and the velocity is expressed by$\dot{p}$. Defining *x*_1_ = *p* and $x_{2}=\dot{p}$, the dynamic equation (5) of the microrobot can be rewritten as the following state equation:

(6) $$\begin{array}{c} \left\{\begin{array}{c} {\dot{\text{x}}}_{1}={\text{x}}_{2},\\ {\dot{\text{x}}}_{2}=b_{0}\text{u}+\frac{1}{m}\left(-{\text{F}}_{\text{drag}}+\Delta \right),\\ y=x_{1}, \end{array}\right. \end{array}$$

where *u* = *F*_*mag*_ and *b*_0_ = 1/*m*.

For the above equation, the total disturbances is defined as *ξ* = (−F_drag_ + Δ)/*m*, it includes the resistance, unmodeled parts, and unknown disturbances during the movement of the microrobot. Obtaining the actual position *p* of the microrobot and total disturbances *ξ*, is the premise of accurate path tracking. The position of the microrobot can be obtained by machine vision, but the total disturbances *ξ* needs to be observed as an extended state by designing ESO. The total disturbances are defined as an extended state, that is, *x*_3_ = *ξ*. Equation (6) can be written as

(7) $$\begin{array}{c} \left\{\begin{array}{c} {\dot{\text{x}}}_{1}={\text{x}}_{2},\\ {\dot{\text{x}}}_{2}=x_{3}+b_{0}\text{u},\\ {\dot{x}}_{3}=\dot{\xi },\\ y=x_{1}. \end{array}\right. \end{array}$$

**Assumption 1 .**

*ξ* is assumed to be continuously differentiable. *ξ*, and $\dot{\xi }$ meet the following conditions: *ξ* ≤ *η*_1_, $\dot{\xi }\leq \eta_{2}$, *η*_1_, and *η*_2_ are positive.

ESO is designed to observe the total disturbances of the system. ESO is built as follows:

(8) $$\begin{array}{c} \left\{\begin{array}{c} {\dot{\text{z}}}_{1}=z_{2}+\beta_{1}e_{1},\\ {\dot{\text{z}}}_{2}=z_{3}+b_{0}u+\beta_{2}e_{1},\\ {\dot{\text{z}}}_{3}=\beta_{3}e_{1},\\ {\text{e}}_{1}=x_{1}-z_{1}, \end{array}\right. \end{array}$$

where *z*_1_, *z*_2__,_ and *z*_3_ are the estimations of the position *x*_1_, velocity *x*_2_, and total disturbances *x*_3_, respectively. *e*_1_ represents position estimation error. *β*_1_, *β*_2_, and *β*_3_ are the observer gains to be chosen.

**Theorem 1 .**

Under the condition of Assumption 1, the observation error of designed ESO is bounded. Selecting appropriate parameters *β*_1_, *β*_2_, and *β*_3_, **z**_1_⟶**x**_1_, **z**_2_⟶**x**_2_, and **z**_3_⟶**x**_3_ as *t*⟶∞.

### 3.3. ESO Convergence Analysis

Let *e* = [*e*_1_ *e*_2_ *e*_3_]^T^, so that $\dot{e}={\left[{\dot{e}}_{1}\;{\dot{e}}_{2}\;{\dot{e}}_{3}\right]}^{\text{T}}$, comprehensive Equations (7) and (8), ${\dot{e}}_{1}$, ${\dot{e}}_{2}$, and ${\dot{e}}_{3}$ can be expressed as

(9) $$\begin{array}{c} \left\{\begin{array}{c} {\dot{\text{e}}}_{1}=e_{2}-\beta_{1}e_{1},\\ {\dot{\text{e}}}_{2}=e_{3}-\beta_{2}e_{1},\\ {\dot{\text{e}}}_{3}=\dot{\xi }-\beta_{3}e_{1}, \end{array}\right. \end{array}$$

where *e*_*i*_ = *x*_*i*_ − *z*_*i*_ (*i* = 1, 2, 3), then the observation error state equation can be written as

(10) $$\begin{array}{c} \dot{e}=Ae+B\dot{\xi }, \end{array}$$

where

(11) $$\begin{array}{c} A=\left[\begin{array}{ccc} -\beta_{1} & 1 & 0\\ -\beta_{2} & 0 & 1\\ -\beta_{3} & 0 & 0 \end{array}\right],\\ B=\left[\begin{array}{c} 0\\ 0\\ 1 \end{array}\right]. \end{array}$$

The characteristic equation of *A* is

(12) $$\begin{array}{c} \left|\lambda I-A\right|=0. \end{array}$$

*β*_*i*_(*i* = 1, 2, 3) should be appropriately selected to make *A* Hurwitz matrix. The parameters can be determined by using the following method:

(13) $$\begin{array}{c} \lambda^{3}+\beta_{1}\lambda^{2}+\beta_{2}\lambda +\beta_{3}={\left(\lambda +\omega_{0}\right)}^{3},\\ =0. \end{array}$$

Consequently, *β*_1_ = 3*ω*_0_, *β*_2_ = 3*ω*_0_^2^, and *β*_3_ = *ω*_0_^3^, where *ω*_0_ is the observation bandwidth.

For any positive definite matrix *Q*, there is a positive definite matrix *P*. The following Lyapunov equation is satisfied

(14) $$\begin{array}{c} A^{T}P+PA=-Q. \end{array}$$

The Lyapunov function of the observer is defined as

(15) $$\begin{array}{c} V_{0}=e^{T}Pe. \end{array}$$

Combining Equation (10), the derivative of the above equation to time is

(16) $$\begin{array}{c} {\dot{V}}_{0}={\dot{e}}^{T}Pe+e^{T}P\dot{e}=e^{T}\left(A^{T}P+PA\right)e+\dot{\xi }\left(B^{T}Pe+e^{T}PB\right)\leq -e^{T}Qe+2\eta_{2}\left\|PB\right\|\cdot \left\|e\right\|. \end{array}$$

According to Equation (16), we have

(17) $$\begin{array}{c} {\dot{V}}_{0}\leq -\lambda_{\min}{\left\|e\right\|}^{2}+2\eta_{2}\left\|PB\right\|\cdot \left\|e\right\|, \end{array}$$

where *λ*_min_ is the minimum eigenvalue of *Q*. To make ${\dot{V}}_{0}\leq 0$, the convergence condition of ESO is

(18) $$\begin{array}{c} e\leq \frac{2\eta_{2}PB}{\lambda_{\min}}. \end{array}$$

To sum up, the designed ESO has convergence.

### 3.4. Controller Design

In this section, the path tracking controller of microrobot will be designed based on sliding mode control. Since the disturbances can be observed by ESO, the disturbances compensation method is used to eliminate the total disturbances. The control law is designed as follows:

(19) $$\begin{array}{c} u=u_{0}-\frac{1}{b_{0}}z_{3}, \end{array}$$

where *b*_0_ = 1/*m* and *z*_3_ is the observation of the total disturbances. As an intermediate control variable, *u*_0_ will be designed based on SMC:

(20) $$\begin{array}{c} u_{0}=u_{eq}+u_{sw}, \end{array}$$

where *u*_*eq*_ is the equivalent control term and *u*_*n*_ is the switching control term. In order to design *u*_0_, the sliding surface is chosen as

(21) $$\begin{array}{c} s=\gamma \hat{e}+\dot{\hat{e}}, \end{array}$$

where *γ* is positive, $\hat{e}$ and $\dot{\hat{e}}$ are the position and velocity tracking error of the microrobot, respectively, which are defined as follows:

(22) $$\begin{array}{c} \left\{\begin{array}{c} \hat{e}=x_{1}-p_{r},\\ \dot{\hat{e}}=x_{2}-{\dot{p}}_{r}, \end{array}\right. \end{array}$$

where *p*_*r*_ represents the desired path of the microrobot. According to Equation (7), by taking the derivative of (20), we have

(23) $$\begin{array}{c} \left\{\begin{array}{c} \dot{\hat{e}}=x_{2}-{\dot{p}}_{r},\\ \ddot{\hat{e}}=b_{0}u+\xi -{\ddot{p}}_{r}. \end{array}\right. \end{array}$$

According to Equation (23), and by taking the derivative of (19), we have

(24) $$\begin{array}{c} \dot{s}=\gamma \dot{\hat{e}}+\ddot{\hat{e}}=\gamma \dot{\hat{e}}+b_{0}u+\xi -{\ddot{p}}_{r}. \end{array}$$

The *u*_*eq*_ can be designed by considering $\dot{s}=0$ and *ξ* = 0. Therefore, *u*_*eq*_ is designed as

(25) $$\begin{array}{c} u_{eq}=\frac{1}{b_{0}}\left({\ddot{p}}_{r}-\gamma \dot{\hat{e}}\right). \end{array}$$

Besides, the switching controller *u*_*sw*_ is adopted as follows:

(26) $$\begin{array}{c} u_{sw}=-\eta \mathrm{sgn}\left(s\right), \end{array}$$

where *η* is the positive switching gain. According to the comprehensive Equations (19), (20), (25), and (26), the path tracking controller of microrobot in complex environment is designed as follows:

(27) $$\begin{array}{c} u=\frac{1}{b_{0}}\left({\ddot{p}}_{r}-\gamma \dot{\hat{e}}\right)-\eta \mathrm{sgn}\left(s\right)-\frac{1}{b_{0}}z_{3}. \end{array}$$

**Figure 3:**
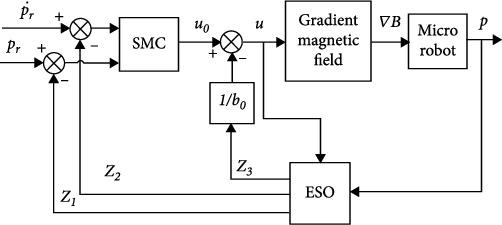
Robust control of microrobot.

**Theorem 2 .**

points out that selecting appropriate parameters *β*_1_, *β*_2__,_ and *β*_3_ of ESO, **z**_1_⟶**x**_1_, **z**_2_⟶**x**_2_, and **z**_3_⟶**x**_3_ as *t*⟶∞. In summary, the robust control block diagram of microrobot in complex environment is shown in Figure 3:

### 3.5. Stability Analysis

The total disturbances observation error is defined as

(28) $$\begin{array}{c} e_{3}=x_{3}-z_{3}\leq \varepsilon . \end{array}$$

Due to the convergence of ESO, *e*_3_ is bounded, and *ε* is the upper bound of *e*_3_. Considering the following Lyapunov function

(29) $$\begin{array}{c} V_{1}=\frac{1}{2}s^{2}. \end{array}$$

The derivative of the above equation to time is

(30) $$\begin{array}{c} {\dot{V}}_{1}=s\dot{s},\\ =s\left(\gamma \dot{\hat{e}}+b_{0}u+\xi -{\ddot{p}}_{r}\right). \end{array}$$

According to Equation (26),

(31) $$\begin{array}{c} {\dot{V}}_{1}=s\left[\left(\xi -z_{3}\right)-b_{0}\eta \mathrm{sgn}\left(s\right)\right]\leq \left|s\right|\left(\varepsilon -b_{0}\eta \right). \end{array}$$

When *η* > *ε*/*b*_0_, we have ${\dot{V}}_{1}=s\dot{s}\leq 0$. According to Lyapunov stability theory, the system is stable under the condition. Therefore, the system states can make the sliding mode variable *s* converge to 0 in a limited time, the position tracking error will also converge to 0.

## 4. Experimental Analysis

**Figure 4:**
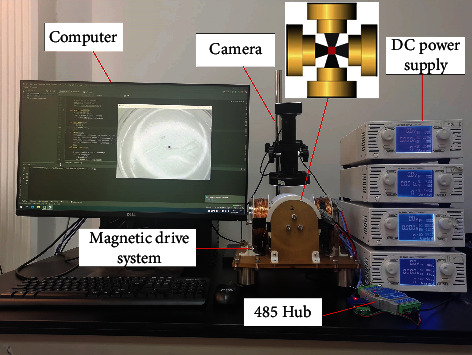
Electromagnetic drive system experiment platform.

In order to verify the effectiveness of the proposed control method, the path tracking control experiments of the magnetic microrobot is carried out in the electromagnetic drive system. The experimental platform is shown in Figure 4. The electromagnetic drive system is composed of four orthogonal electromagnetic coils with the same parameters, the workspace size is 16 mm × 16 mm. The electromagnetic coils are composed of soft magnetic core and coils. The soft magnetic core designed by ourselves is used to enhance the magnetic flux density and magnetic field gradient. The electromagnetic coils are supplied with current by programmable DC power supply. When the maximum output current is 5 A, it can produce a magnetic flux density of 73.93 mT and a gradient of 8.68 T/m in the center of workspace. In the experiments, a spherical magnetic microrobot with a diameter of 300 *μ*m and a density of 1.3 × 10^3^ kg/m^3^ is selected as the driving object. The moving medium of the microrobot is silicone oil solution. CCD industrial camera is used to obtain the real-time position of the microrobot and realize visual feedback. The control algorithm is written by Python language on the computer, and the computer communicates with the DC power supply through 485 bus.

The path tracking process of microrobot is generally as follows: a path with starting point and ending point is set in a simulated vascular structure, and the microrobot is placed near the starting point. When the path tracking control experiments is carried out, the microrobot moves to the starting point and then completes the tracking of the whole path.

**Figure 5:**
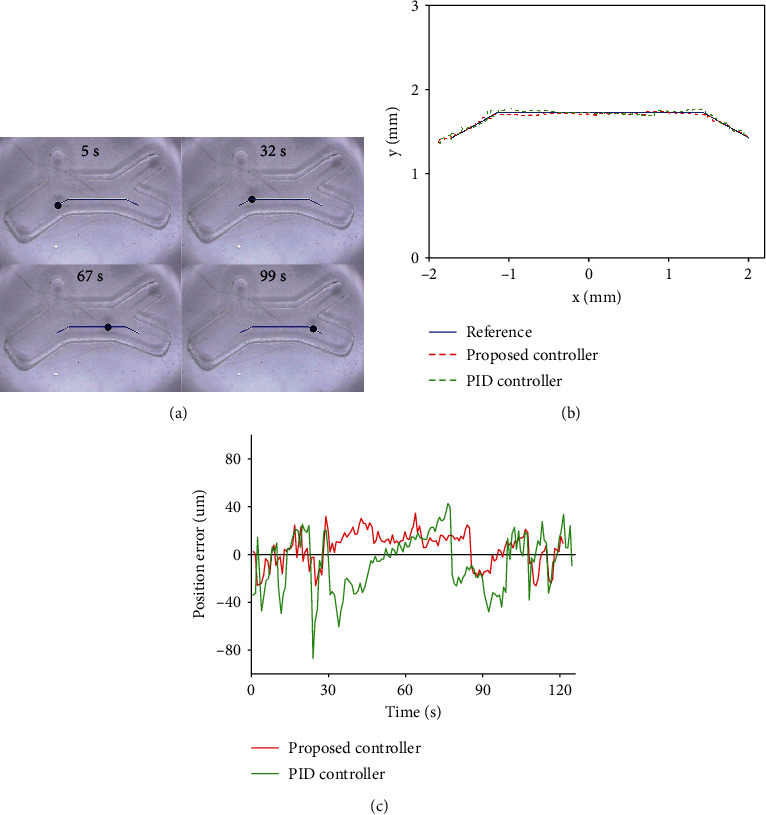
Path 1 tracking results of microrobot: (a) tracking processes; (b) experiment results; and (c) tracking errors.

Silicone oil solution with viscosity of 50 CS was selected as the motion medium of microrobot. In the experiments, the traditional PID control was used as the comparison method to reflect the superiority of the control method proposed in this paper. In the first experiment, the reference path of the microrobot was set as a curve from left to right in a simulated vascular structure, hereinafter referred to as path 1.  Figure 5(a) shows the different stages of the microrobot tracking path 1.  Figure 5(b) shows the path tracking experimental results under two control methods, and Figure 5(c) shows the path tracking errors of the microrobot. The error range, mean absolute error (MAE), and root mean square error (RMSE) are shown in Table 1.

According to Table 1, using the robust control method based on ESO, the error range of the microrobot tracking path 1 is -32.46~37.75 *μ*m. The mean absolute error is 13.30 *μ*m. The root mean square error is 15.39 *μ*m. The results is obviously lower than the path tracking errors of the microrobot under the traditional PID control method.

**Figure 6:**
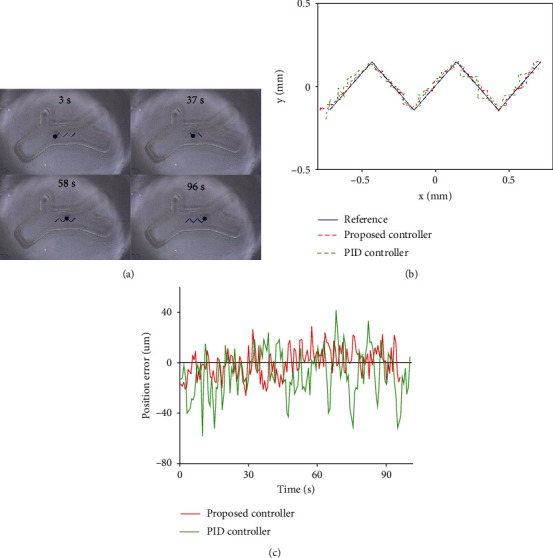
Path 2 tracking results of microrobot: (a) tracking processes; (b) experiment results; and (c) tracking errors.

In the second experiment, two control methods were used to track the sawtooth path, hereinafter referred to as path 2.  Figure 6(a) shows the different stages of microrobot tracking path 2.  Figure 6(b) shows the experimental results of the microrobot tracking path 2 under the control method proposed in this paper and the traditional PID control. The tracking errors are shown in Figure 6(c). The error range, mean absolute error, and root mean square error are shown in Table 2.

According to the analysis of Table 2, using the robust control method based on ESO, the error range of microrobot tracking path 2 is -27.45~32.02 *μ*m. The mean absolute error is 10.02 *μ*m. The root mean square error is 12.27 *μ*m. Compared with the traditional PID control method, it has smaller tracking errors.

**Figure 7:**
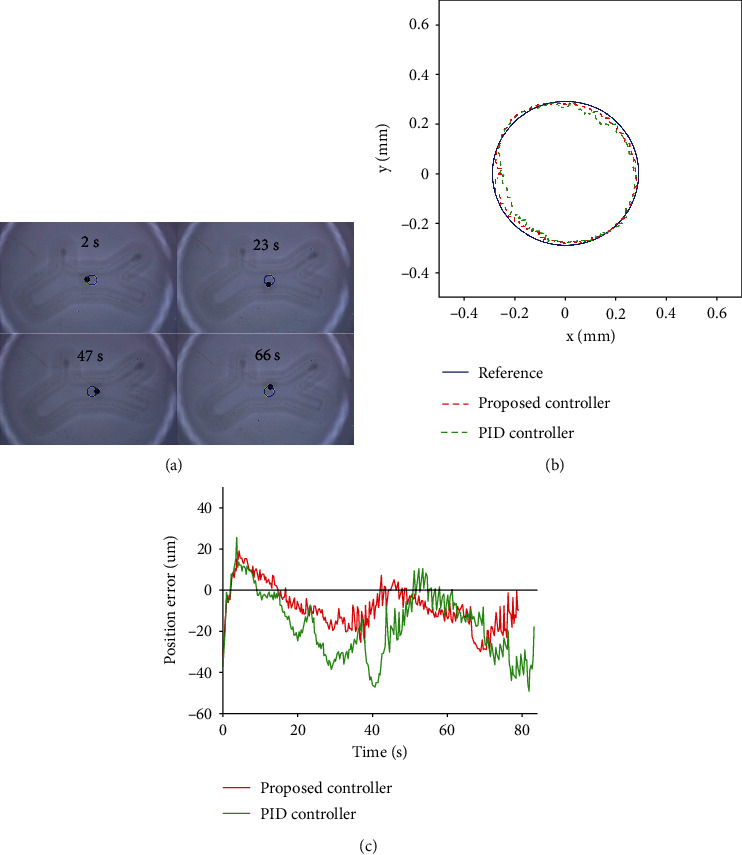
Circular path tracking results of microrobot: (a) tracking processes; (b) experiment results; and (c) tracking errors.

In the third experiment, a circular path was set in the center of the simulated vascular structure for the tracking control of microrobot.  Figure 7(a) shows the different stages of microrobot tracking the circular path. The experimental results of the microrobot tracking circular path under the control method proposed in this paper and the traditional PID control are shown in Figure 7(b).  Figure 7(c) reflects the tracking errors of the whole process. The error range, mean absolute error, and root mean square error are shown in Table 3.

According to the analysis of Table 3, the error range of microrobot tracking circular path is -33.64~19.06 *μ*m by using the robust control method proposed in this paper. The mean absolute error is 11.10 *μ*m. The root mean square error is 13.11 *μ*m. This method makes the microrobot track the circular path more accurately.

In the research process, to avoid the contingency of the experimental results and reflect the persuasiveness of the control algorithm proposed in this paper, the path tracking control experiments of the microrobot under the above three paths in a simulated vascular structure were carried out for many times.  Table 4 lists multiple groups of path tracking error data by using robust control method based on ESO and traditional PID control.

To sum up, the robust control method based on ESO proposed in this paper can successfully realize the path tracking control of microrobot in the complex environment with a variety of dynamic disturbances. Furthermore, compared with the traditional PID control, the proposed control method show better tracking performance.

## 5. Conclusion

A robust control method based on ESO is proposed in this paper to realize the precise path tracking control of microrobot. Firstly, the dynamic model of the microrobot in the gradient magnetic field is analyzed, and then the path tracking control method of the microrobot in the complex environment is designed based on this model. The proposed method takes the uncertainties of system model parameters and external environmental interference as the total disturbances, which can be observed by ESO and eliminated by disturbances compensation strategy. At the same time, sliding mode control is used to suppress the position error of microrobot and ensure the robustness of tracking. To verify the effectiveness of the control method, the experiments of microrobot tracking two different paths in a simulated vascular structure are carried out. The experimental results show that the proposed control method can successfully realize the path tracking control of the microrobot and has satisfactory tracking accuracy. Therefore, the method is robust to unknown disturbances and the uncertainties of system parameters in complex environment. In the following research, with the hardware conditions of in vivo imaging, we can try to apply this control method to real living biological vascular.

## Figures and Tables

**Table 1:** Comparison of path 1 tracking errors of microrobot under two control methods.

Path 1	PID control	Proposed
Error range (*μ*m)	-88.09~45.19	-32.46~37.75
MAE (*μ*m)	20.60	13.30
RMSE (*μ*m)	25.17	15.39

**Table 2:** Comparison of path 2 tracking errors of microrobot under two control methods.

Path 2	PID control	Proposed
Error range (*μ*m)	-59.17~41.53	-27.45~32.02
MAE (*μ*m)	16.48	10.02
RMSE (*μ*m)	20.78	12.27

**Table 3:** Comparison of circular path tracking errors of microrobot under two control methods.

Circular path	PID control	Proposed
Error range (*μ*m)	-49.20~25.75	-33.64~19.06
MAE (*μ*m)	18.73	11.10
RMSE (*μ*m)	22.69	13.11

**Table 4:** Multiple groups of path tracking errors.

Experiment no.	Path	Control method	MAE (*μ*m)	RMSE (*μ*m)	Error range (*μ*m)
1	Path 1	Proposed	13.79	15.93	-38.15~29.06
2			13.92	16.13	-40.03~31.43
3			13.43	15.84	-25.78~36.37
4		PID control	21.39	27.16	-65.14~46.11
5			20.96	25.88	-70.76~48.70
6			21.22	27.31	-73.22~50.91

7	Path 2	Proposed	10.86	13.08	-25.69~36.25
8			11.43	13.76	-29.85~31.09
9			11.15	14.19	-31.03~32.34
10		PID control	17.27	21.09	-59.48~41.18
11			17.03	21.38	-62.09~39.47
12			16.94	20.97	-48.37~44.37

13	Circular path	Proposed	12.42	14.03	-37.61~20.88
14			12.94	14.85	-27.50~28.13
15			11.81	13.92	-30.47~24.49
16		PID control	18.24	21.91	-52.32~28.62
17			19.67	23.22	-47.86~31.77
18			19.24	23.89	-64.07~29.35

## Data Availability

The data used to support the findings of this study are included within the article.
